# Climatic factors, but not geographic distance, promote genetic structure and differentiation of Cleistogenes squarrosa (Trin.) Keng populations

**DOI:** 10.3389/fbinf.2024.1454689

**Published:** 2024-11-13

**Authors:** Ruyan Song, Xueli Zhang, Zhuo Zhang, Chan Zhou

**Affiliations:** ^1^ School of Life Science, Liaoning University, Shenyang, China; ^2^ School of Life Science and Bioengineering, Shenyang University, Shenyang, China

**Keywords:** genetic diversity, genetic structure, Cleistogenes squarrosa Keng, climatic factors, geographic distance

## Abstract

Climate can shape plant genetic diversity and genetic structure, and genetic diversity and genetic structure can reflect the adaptation of plants to climate change. We used rbcl and trnL-trnF sequences to analyze the genetic diversity and genetic structure of *C. squarrosa* under the influence of different environmental factors in Inner Mongolia grassland. The results showed that the genetic diversity of this species was low. (The trnL-trnF sequences have higher genetic diversity than rbcl sequences.) *C. squarrosa* had low genetic diversity compared to other prairie plants, but had a more pronounced genetic structure. The haplotype network diagram of the combined sequences could be divided into two categories, and the results of the NJ, MP, and ML trees also showed that the haplotypes were divided into two branches. The results of genetic structure analysis showed that that the populations located in the desert steppe fall into exactly one cluster, and the populations located in the typical steppe fall into exactly another cluster. The neutrality tests were all negative and the mismatch distribution also showed a single peak across the population, suggesting that *C. squarrosa* had undergone population expansion and was well adapted to the local environment. The results of the mantel test showed that climate had a greater influence on the genetic distance of *C. squarrosa*, with annual precipitation having a higher influence than mean annual temperature. This study provided basic genetic information on the genetic structure of *C. squarrosa* and contributes to the study of genetic adaptation mechanisms in grassland plants.

## 1 Introduction

Genetic diversity is critical to population survival and evolution, especially in changing environments (Santelices et al., 2018). Genetic diversity arises from mutations in the DNA sequence of cell lines, and from there, it reaches the level of organisms, populations, and regions (Hughes et al., 2008; Engelhardt et al., 2014). At the organism level, genetic diversity includes genetic variation at the gene level. At the population level, genetic diversity is measured by allelic and genotypic diversity, including the number of alleles and genotypes found in a population and their relative abundance. Populations with higher genotypic diversity exhibit higher productivity, higher resistance to pathogens, and a better capacity to recover after environmental disturbances (Bell, 1991). At the regional level, genetic diversity is measured by comparisons between populations, which are expected to have gone through local adaptation and thus show genetic differentiation (Kawecki and Ebert, 2004). From an ecological perspective, genetic diversity is usually considered as an advantage since there are positive correlations between genetic diversity, fitness, and population survival (Dole and Sun, 1992; Buza et al., 2000). From an evolutionary viewpoint, genetic diversity is needed for population adaptations in changing environments (Booy et al., 2000; Reed and Frankham, 2003). Previous studies have focused on the relationship between genetic diversity and evolution, mostly on small spatial scales, but the relationship between genetic diversity and the climate on large-scale gradients has been less well investigated (Lin et al., 2006).

Environmental factors and habitat heterogeneity have a great role in shaping the genetic diversity of species (Kabiel et al., 2013). Within populations, genetic diversity is considered to be essential for adaptations to environmental change (Hamasha et al., 2013), consequently, for the long-term survival of plant populations (Słomka et al., 2011). Strong environmental gradients can affect the genetic structure of plant populations and may lead to limited gene flow between populations, ultimately enhancing genetic variation between populations (Hamasha et al., 2013). The climate is one of the most important drivers of local adaptation in plant species; climate change has the potential to alter the genetic diversity of plant populations with consequences for community dynamics and ecosystem processes (Huang et al., 2016). Changes in climate affect plant phenological patterns (e.g., flowering time and seed germination) and mating systems (e.g., pollen dispersal), thereby increasing selection pressure on populations between different regions and affecting the genetic structure of populations (Hamasha et al., 2013).

Temperature and precipitation are among the main environmental factors affecting genetic differentiation and diversity among populations (Al-Gharaibeh et al., 2017). The influence of temperature on genetic variation is mainly reflected in plant phenology (Liu et al., 2020). For example, temperature changes affect the timing of plant flowering, variation in flowering phenology can potentially increase reproductive isolation and genetic differentiation within and among populations, affecting genetic differentiation within and between populations and leading to the formation of genetic structure (Luo et al., 2021). Hence, early-flowering plants are likely to mate with other early-flowering plants, while late-flowering plants are likely to mate with other late-bloomers (Pramuk and Runkle, 2005). The effects of precipitation on genetic diversity are more often seen in the mating systems of plants. Particularly for water-borne pollinator species, the pattern of variation is determined by precipitation, which in turn affects seed dispersal (Kabiel et al., 2013). Located on the northern frontier of China, the Inner Mongolia Autonomous Region has a large area and a longitude span (Cui D. et al., 2021). It is characterized by a typical mid-temperate continental monsoon climate with dramatic intra-annual temperature variation and uneven precipitation distribution (Naugžemys et al., 2022). Differences in precipitation and temperature allow for robust analysis of the composition of genetic variation in populations across large-scale gradients (Ying et al., 2020).

Chloroplast genomes are highly conserved among species and are often used to analyze interspecific genetic diversity (Shinozaki et al., 1986). CUI et al. demonstrated high genetic diversity among melon populations from the perspective of chloroplast genes (Cui H. et al., 2021). It is also demonstrated that the decrease in genetic diversity in melon had a significant effect on the variety of the chloroplast genome (Cui et al., 2020). However, there needs to be more in-depth studies on the impact of chloroplasts on genetic diversity over large-scale gradients (Leimu et al., 2006). The insertion sequences in the chloroplast trnL-trnF sequences are highly variable and informative for inter-species differentiation studies, while the rbcl sequences are relatively conserved and suitable for low-level phylogenetic analyses and population structure studies within species. The simultaneous use of these two sequences can improve the resolution and accuracy of genetic analysis. Therefore, these two fragments were selected for analysis in this paper, so as to effectively evaluate the polymorphism level and population expansion pattern of *C. squarrosa* populations.

Plants cannot escape environmental stress and are fully exposed to a variety of environments during their life cycle. They have developed many adaptive mechanisms to survive in a constantly changing environment. The Cleistogenes squarrosa Keng is widely distributed in the Eurasian steppe zone. In China, it is mainly found in the typical grasslands, meadows, and desert grasslands of the Inner Mongolian Plateau. It is the dominant lower-layer species in needlegrass grasslands and sheep grass grasslands. *C. squarrosa* is a small perennial tufted grass of the genus Cleistogenes, highly resistant to cold, with spikes around mid-July and flowering usually in early to mid-August. Temperature affects the return of *C. squarrosa*, while precipitation will have a facilitative effect on the tassel and flowering stages (Zhang et al., 2014). As a significantly associated species of typical grasslands in Inner Mongolia, *C. squarrosa* is highly adaptable to adapt environment change. In terms of morphology, when precipitation decreases, the specific leaf area of C. squarrosa decreases. Leaf nitrogen content per unit area increases significantly, indicating that *C. squarrosa* adapts to drought environments by changing morphological and physiological traits when precipitation changes (Zhang et al., 2019). At the same time, the root depth of C. squarrosa is around 10 cm, which enables it to cope with environments with low precipitation. Qi Yan et al. explored the response of Cleistogenes songorica to drought stress at the gene level and found that increase, miRNAs, PCgenes, and transcription factors together constitute a complex transcriptional regulatory system to adapt to water stress (Zhang et al., 2020). As a typical C4 plant, C. squarrosa is more adaptable to environmental changes than other grassland plants and can be assessed for climate change in Inner Mongolian grasslands (Zhang et al., 2014). Therefore, using *C. squarrosa* as experimental material to investigate the relationship between environmental change and genetic diversity is more appropriate (McRae, 2006).

The association between genetic and climatic gradients has been well-established as evidence of natural selection (Manel et al., 2010). In the present study, 20 sites of *C. squarrosa* in the Inner Mongolia grassland were used as experimental samples, and these sample sites showed gradients in precipitation and temperature. Studies over such environmentally heterogeneous geographic ranges are promising to reveal the influence of climatic conditions on plant genetic structure (Nevo, 2001; Hamasha et al., 2013). In this study we will illustrate the following points: (1) the effects of climate factors on the genetic diversity and genetic structure of *C. squarrosa* between different geographical regions; and (2) the genetic differentiation characteristics of *C. squarrosa* in 20 sample sites. This study revealed the information underlying the genetic diversity and structure of *C. squarrosa*, and provided a theoretical basis for the genetic adaptation mechanisms of *C. squarrosa*.

## 2 Materials and methods

### 2.1 Study area

**FIGURE 1:**
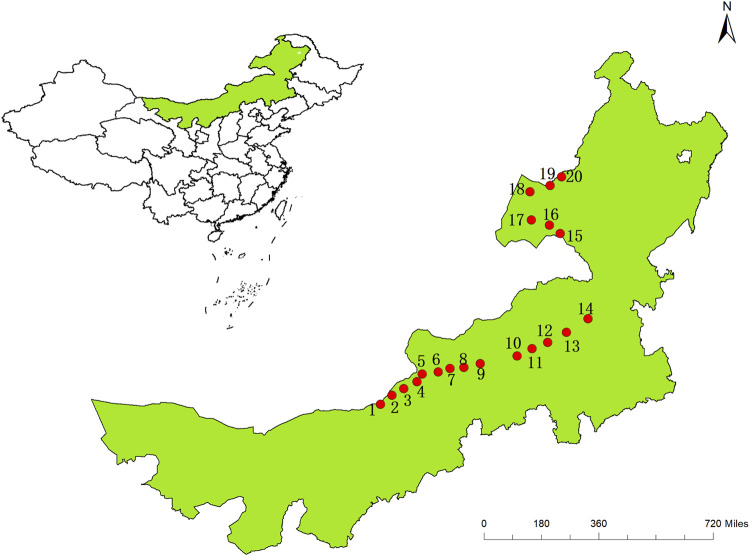
Distribution of sampling points.

This study was conducted along an east-west transect of 3,000 km in northern China’s arid and semi-arid grasslands, mainly in northeastern Inner Mongolia. The sample zone has a longitude range of 110.294–118.533 ºE and a latitude range of 42.623º–49.783° N. The sample zone has a typical continental climate with limited summer precipitation. Along the sample zone, mean annual precipitation (AP) increases from 139 mm in the west to 443 mm in the east, and annual mean temperature (MAT) ranges from a maximum of 6.3°C to a minimum of −0.1°C (AP and MAT data from the worldclim website) (Table 1). Thus the main vegetation types from west to east are desert steppe and typical steppe, and the main soil types are black calcareous soil, chestnut calcareous soil, gray meadow soil, and gray forest soil. Of the C. squarrosa collected in this study, populations 1 to 8 are in the desert steppes, and 9 to 20 are in the typical steppes (Figure 1). The 20 samples selected, running through the east-west sample zone of our study area and with each population being representative of its region, provide a good reflection of the influence of climatic factors on the genetic structure of populations.

**TABLE 1:** Basic information of sample points.

Locations_ID	Sample range	Latitude (°N)	Longitude (°E)	AP (mm)	MAT (°C)
1	13–19	42.623956	110.294922	151	6.3
2	3–9	42.931872	110.824219	157	6.3
3	16–19	43.147136	111.355072	131	5.8
4	9–18	43.381808	111.964256	146	5.4
5	15–18	43.634756	112.196397	139	5.3
6	16–19	43.707139	112.921311	175	4.4
7	14–17	43.818969	113.466339	192	4.2
8	8–16	43.849617	114.085617	190	3.9
9	18–19	43.979447	114.826747	239	3.0
10	6–11	44.221517	116.507064	332	2.6
11	19–20	44.466583	117.179733	330	2.4
12	7–20	44.666186	117.895758	401	1.5
13	5–20	44.989969	118.745803	404	1.7
14	17–20	45.426797	119.723061	443	1.3
15	19	48.088833	118.455567	307	0.5
16	13–18	48.344544	117.979492	261	1.3
17	18	48.497028	117.151989	279	1.2
18	12–15	49.338222	117.091178	299	−0.1
19	8–12	49.529275	118.009956	370	0
20	18–19	49.783856	118.533464	320	0

### 2.2 Plant sampling and DNA extraction

Samples were collected from 2012 to 2015 and frozen after collection. The experimental material for this experiment was taken from the young leaves of *C. squarrosa* seedlings at the above sample sites and the total DNA were extracted from the chloroplasts of these young leaves by CTAB method, followed by the purity of DNA was determined by 1% agarose gel electrophoresis, and a spectrophotometer was selected to determine its concentration, and finally placed at −20°C for backup.

The trnL-trnF primers used for PCR amplification were trnL (TabC) (5-CGAAATCGGTAGACGCTACG-3′), and trnF (TabF) (5′-ATTTGAACTGGTGACACGAG-3′), the upstream primer for rbcl was (NcoI) (5′- CATGCCATGGTTATGTCAC- CACAAACAGAGA-3′), and the downstream primer was (XhoI) (5′-CCGCTCGAGTTAG- GAAAAGATTGTGCCGAG-3′). The PCR amplification system included 1 μL of template DNA, 0.5 μL of upstream primer, 0.5 μL of downstream primer, 0.5 μL (10 mmol-L-1) of dNTP, 2.5 μL of 10×buffer, 2.0 μL (25 mmol-L-1) of Mg2+, 0.2 μL (5 U-μL-1) of Taqase, 17.8 μL of ddH2O for a total of 25 μL of amplification system. The PCR amplification system was set up as follows: pre-denaturation at 95°C for 3 min; denaturation at 95°C for 30 s, annealing at 60°C for 30 s, and extension at 72°C for 30 s for 10 cycles; denaturation at 95°C for 30 s, annealing at 55°C for 30s, and extension at 72°C for 30 s. This process was carried out for 20 cycles, and finally, extension at 72°C for 6 min. The amplified products were detected by electrophoresis using a DNA sequencer and the peak electrophoresis profiles of all amplified products were obtained for subsequent analysis. Taq enzyme from Thermo Fisher, Sequencers were performed using the Thermo Fisher Applied Biosystems TM 3730XL sequencer. The above experimental conditions are the best conditions for PCR amplification after several pre-experiment. Currently, the four sequences suitable for plant genetic diversity analysis are rbcl, ITS, trnL-F, matk, of which ITS and matk are more suitable for genetic evolution analysis, and rbcl and trnL-F are more suitable for genetic evolution analysis which is more in line with the research content of this paper.

### 2.3 Data analysis

Peak electrophoresis profiles were converted to amplified fragments using GeneMapper, and the data were imported into CLUSTALX for sequence comparison and manual correction. Nucleotide polymorphism (Pi), the mean number of nucleotide differences (K), haplotype diversity (Hd), number of haplotypes (h), random deviation of haplotype diversity (Sh), Tajima’s D and Fu’s Fs values were calculated using DNAsp (https://doi.org/10.1093/molbev/msx248) (Liu et al., 2020). And haplotype network structure analysis by NETWORK. The variance between and within populations was estimated using molecular analysis of variance (amova) using 1,000 bootstrap. Genetic distance and Fst values between and within populations were calculated using MEGA 11 (https://doi.org/10.1093/molbev/msab120). Genetic distances were estimated using variance estimation, selected by the p-distance method. The NJ tree was obtained by heuristic search using PAUP4.0 software, with default settings selected for the multitree parameters, while 1,000 bootstrap bootstrap tests were performed. The non-coercive topological tree shapes of equally distant trees were compared using the kishino-hasegawa likelihood test, and the best topology (min-nl) was chosen. Maximum Parsimony (MP) trees are also calculated using PAUP 4.0 (https://api.semanticscholar.org/CorpusID:90795438) software, while Maximal likelihood (ML) trees are calculated using IQtree software. ArcGIS software was used to map the haplotype distribution of the populations (Peleg et al., 2008).

To determine the population structure, Structure 2.3 was used for Bayesian clustering analysis (Cui D. et al., 2021). This model has both the analytical advantages of a Bayesian model over other models and the ability to analyze the clustering of individuals in groups. The program used a mixed model and independent allele frequencies using MCMC replication. Ten independent operations were performed for each of the classifications from 1 to 19. The burn-in length was 20,000, and run length was 1,200,000 and final k values were determined based on LnP(D) and Δk in the output of structure results (Cui D. et al., 2021).

Bioclimatic variables for each sample site were taken from the worldclim website. These data are available in four spatial resolutions, ranging from 30 s (∼1 km^2^) to 10 min (∼340 km^2^) (Fick and Hijmans, 2017). Nineteen climatic variables were extracted for each site from 1970–2000 using the R package (https://doi.org/10.1016/j.envsoft.2018.09.009). Principal component analysis (PCA) was performed on the 19 bioclimatic variables using the FactoMineR (https://doi.org/10.18637/jss.v025.i01) package (Table 2). Annual precipitation and annual mean temperature were selected as the main units of analysis for the climatic variables. The mantel and partial mantel tests were performed using the vegan package in R (https://doi.org/10.1111/j.1654-1103.2003.tb02228.x) to assess. The relationship between genetic and geographic distances and environmental distances was assessed, and 9,999 permutations were set (Haider et al., 2012). Setting the number of permutations to 9,999 ensures the accuracy of our calculations.

**TABLE 2:** 19 bioclimatic factors used in this study.

Code	Bioclimatic factor	Abbreviation
1	Annual Mean Temperature	MAT
2	Mean Diurnal Range	MDR
3	Isothermality	I
4	Temperature Seasonality	TS
5	Max Temperature of Warmest Month	MTWM
6	Min Temperature of Coldest Month	MTCM
7	Temperature Annual Range	TAR
8	Mean Temperature of Wettest Quarter	MTWQ
9	Mean Temperature of Driest Quarter	MTDQ
10	Mean Temperature of Warmest Quarter	MTW
11	Mean Temperature of Coldest Quarter	MTC
12	Annual Precipitation	AP
13	Precipitation of Wettest Month	PWM
14	Precipitation of Driest Month	PDM
15	Precipitation Seasonality	PS
16	Precipitation of Wettest Quarter	PWQ
17	Precipitation of Driest Quarter	PDQ
18	Precipitation of Warmest Quarter	PW
19	Precipitation of Coldest Quarter	PC

## 3 Results

### 3.1 Genetic diversity

The selected chloroplast gene regions of C. squarrosa differed from each other, and the levels of population genetic diversity varied considerably (Table 3). The trnL-trnF sequence had a relatively high level of polymorphism with a nucleotide polymorphism (pi) of 0.00544, and an average nucleotide difference (K) of 3.50403. The rbcl sequence had the next highest level of polymorphism with a nucleotide polymorphism (pi) of 0.00117, and a mean nucleotide difference (K) of 1.52028. The lowest level of polymorphism was observed after combining fragments from both regions, with nucleotide polymorphisms and average nucleotide differences of 0.00096 and 1.24500, respectively.

**TABLE 3:** The genetic diversity and haplotype diversity analysis in *C. squarrosa* based on 1 non-coding fragment *trnL-trnF* and a conserved coding fragment *rbcl*.

Fragment	Nucleotide Diversity	Average number of nucleotide difference	Haplotype diversity	Number of haplotype	Variance of haplotype diversity	Standard deviation of haplotype diversity	Neutrality and statistical significance	
	(p_i_)	(K)	(Hd)	(h)	(vh)	(Sh)	*Tajima’s D*	*Fu’s Fs*
*rbcl*	0.00117	1.52028	0.561	19	0.00025	0.016	−2.65547***	−8.171
*trnL-trnF*	0.00544	3.50403	0.514	9	0.00025	0.016	−2.64451***	4.887
Combind sequence	0.00096	1.24500	0.544	15	0.00035	0.019	−2.75509***	−6.196

Note: (*) mean *p* < 0.05, (**) mean *p* < 0.01, (***) mean *p* < 0.001.

**FIGURE 2:**
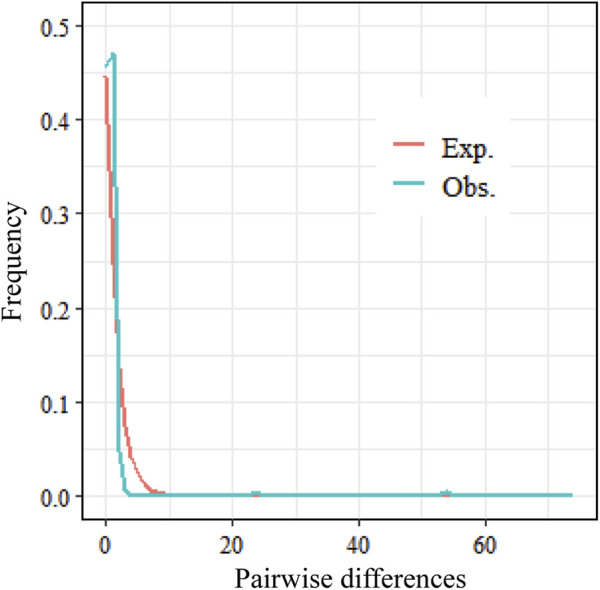
Mismatch distribution established for *C. squarrosa*: the red line represents the expected mismatch distribution of a stationary population. Note: The green line represents the observed mismatch distribution from segregating sites of the aligned sequences of *rbcl* and *trnL-trnF* intergenic spacers of the chloroplast DNA in *C. squarrosa*.

The Tajima’s D values for both coding regions were negative and highly significantly different (P < 0.001) (Table 3), and the Tajima’s D and Fu’s Fs values for the rbcl sequence were lower than those for the trnL-trnF sequence, at −2.65547 and −8.171, respectively. The combined sequence had the smallest Tajima’s D value at −2.75509 and the Fu’s Fs value at −6.196, which were also significantly different (P < 0.001). Tajima’s D and Fu’s Fs values were lower than those of the trnL-trnF sequence, at −2.65547 and −8.171, respectively, while Tajima’s D was the smallest at −2.75509 and Fu’s Fs at −6.196, which were also significantly different (P < 0.001). It is hypothesized that *C. squarrosa* may have undergone population expansion or purifying selection during its evolution, following a neutral evolutionary pattern, and the genetic differentiation was all significantly different. The above negative results indicate that the population faced environmental pressures during evolution but still had partially evolved populations, suggesting that climatic factors had an effect on the genetic structure of the population. The results of the mismatch distribution showed a unimodal distribution, which also proved that the population of Cleistogenes squarrosa (Trin.) Keng experienced population expansion under selective pressure. Combined with the results of the neutrality test and the mismatch distribution, it can be seen that the population of Cleistogenes squarrosa (Trin.) Keng has experienced a relatively smooth expansion and has not undergone rapid expansion in the recent past (Figure 2).

All populations’ rbcl and trnL-trnF sequences were combined and analysed for genetic diversity. The nucleotide polymorphism (pi), mean nucleotide difference (K), haplotype diversity (Hd), and haplotype distribution of the various populations are shown in Table 4. There were significant differences in polymorphism between the populations after combining sequences. Populations 7 and 9 had the highest nucleotide polymorphism of 0.00775 and 0.00728, respectively, and hence the highest mean nucleotide differences of 17.286 and 16.2222 for populations 7 and 9, respectively. Populations 2, 3, 4, 5, 6, 8, 11, 12, 13, 14, and 20 all had zero nucleotide polymorphisms.

**TABLE 4:** The genetic diversity and haplotype diversity analysis of different population in C. squarrosa based on combined sequence.

Population	Nucleotide diversity (Pi)	Average number of nucleotide differences (K)	Haplotype diversity (Hd)	Number of haplotype (h)	Distribution of haplotype
1	0.00007	0.154	0.154	2	Hap1 (12), Hap5 (1)
2	0	0	0	1	Hap1 (3)
3	0	0	0	1	Hap1 (16)
4	0	0	0	1	Hap1 (9)
5	0	0	0	1	Hap1 (15)
6	0	0	0	1	Hap1 (16)
7	0.00775	17.286	0.396	4	Hap1 (11), Hap2 (1) Hap3 (1), Hap15 (1)
8	0	0	0	1	Hap1 (8)
9	0.00728	16.22222	0.111	2	Hap4 (17), Hap9 (1)
10	0.00045	1.00000	0.600	3	Hap4 (4), Hap10 (1) Hap14 (1)
11	0	0	0	1	Hap4 (19)
12	0	0	0	1	Hap4 (7)
13	0	0	0	1	Hap4 (5)
14	0	0	0	1	Hap4 (17)
15	0.00008	0.10526	0.105	2	Hap4 (18), Hap11 (1)
16	0.00007	0.15385	0.154	2	Hap4 (12), Hap7 (1)
18	0.00032	0.66667	0.455	4	Hap4 (9), Hap6 (1) Hap7 (1), Hap13 (1)
19	0.00034	0.75000	0.464	3	Hap4 (6), Hap8 (1) Hap12 (1)
20	0	0	0	1	Hap4 (18)

The results of AMOVA molecular variation analysis showed that the genetic variation among the 19 geographic populations of *C. squarrosa* was 59.17%, and within each geographic population was 40.83%, indicating that variation in *C. squarrosa* occurs both among and within geographic population species, and the difference between the two is negligible (Table 5).

**TABLE 5:** Analysis of molecular variance (AMOVA) within and among population.

Source of variation	df	Variance component	Percentage of total variance	Φst	*p*
Among populations	18	1.48102Va	59.17	0.5917	<0.001
Within populations	216	1.02211Vb	40.83	0.4083	<0.001
Total	234	2.50314			

The genetic distances of the various populations of *C. squarrosa* varied widely (Table 6). Populations 7 and 8 had the most significant genetic distance of 0.01. Population 7 also had a relatively large genetic distance of 0.006–0.007 from populations 10, 11, 12, 13, 14, 15, 16, 18, 19, and 20. Population 9 had a genetic distance of 0.006 from populations 1, 2, 3, 4, 5, 6, and 8, with the remaining populations having a relatively small genetic distance. The first values also differed more markedly between populations. 11, 12, 13, 14, 20 populations and 2, 3, 4, 5, 6, and 8 populations all had a genetic differentiation index of 1, indicating that these populations were completely isolated. Genetic differentiation indices between the remaining populations ranged from −0.191 to 0.954 and were more or less genetically similar.

**TABLE 6:** Genetic distance and pairwise fixation indices Fst (upper diagonal) of genetic variation of different population in *C. squarrosa* based on combined sequence.

Population	1	2	3	4	5	6	7	8	9	10	11	12	13	14	15	16	18	19	20
1	—	−0.191	0.017	−0.031	0.011	0.017	0.000	−0.042	0.316	0.842	0.990	0.983	0.981	0.989	0.940	0.905	0.822	0.870	0.990
2	0.000	—	0.000	0.000	0.000	0.000	−0.191	0.000	0.133	0.691	1.000	1.000	1.000	1.000	0.925	0.861	0.724	0.772	1.000
3	0.000	0.000	—	0.000	0.000	0.000	0.017	0.000	0.345	0.875	1.000	1.000	1.000	1.000	0.954	0.924	0.848	0.898	1.000
4	0.000	0.000	0.000	—	0.000	0.000	−0.031	0.000	0.272	0.818	1.000	1.000	1.000	1.000	0.942	0.900	0.802	0.855	1.000
5	0.000	0.000	0.000	0.000	—	0.000	0.011	0.000	0.336	0.869	1.000	1.000	1.000	1.000	0.952	0.922	0.843	0.893	1.000
6	0.000	0.000	0.000	0.000	0.000	—	0.017	0.000	0.345	0.875	1.000	1.000	1.000	1.000	0.954	0.924	0.848	0.898	1.000
7	0.004	0.004	0.004	0.004	0.004	0.004	—	−0.042	0.307	0.743	0.916	0.866	0.851	0.910	0.872	0.826	0.751	0.776	0.913
8	0.000	0.000	0.000	0.000	0.000	0.000	0.004	—	0.258	0.805	1.000	1.000	1.000	1.000	0.939	0.895	0.792	0.846	1.000
9	.0.006	0.006	0.006	0.006	0.006	0.006	0.010	0.006	—	−0.054	0.003	−0.066	−0.103	−0.003	0.002	−0.018	−0.011	−0.040	0.000
10	0.003	0.003	0.003	0.003	0.003	0.003	0.007	0.003	0.004	—	0.357	0.140	0.071	0.331	0.187	0.048	−0.079	−0.069	0.344
11	0.003	0.003	0.003	0.003	0.003	0.003	0.006	0.003	0.004	0.000	—	0.000	0.000	0.000	0.000	0.030	0.161	0.244	0.000
12	0.003	0.003	0.003	0.003	0.003	0.003	0.006	0.003	0.004	0.000	0.000	—	0.000	0.000	−0.067	−0.055	0.038	0.074	0.000
13	0.003	0.003	0.003	0.003	0.003	0.003	0.006	0.003	0.004	0.001	0.001	0.001	—	0.000	−0.104	−0.096	−0.007	0.018	0.000
14	0.003	0.003	0.003	0.003	0.003	0.003	0.007	0.003	0.004	0.001	0.000	0.000	0.001	—	−0.006	0.021	0.146	0.223	0.000
15	0.003	0.003	0.003	0.003	0.003	0.003	0.006	0.003	0.004	0.000	0.000	0.000	0.001	0.000	—	−0.024	0.066	0.082	−0.003
16	0.003	0.003	0.003	0.003	0.003	0.003	0.007	0.003	0.004	0.000	0.000	0.000	0.001	0.001	0.000	—	−0.015	0.011	0.026
18	0.003	0.003	0.003	0.003	0.003	0.003	0.007	0.003	0.004	0.000	0.000	0.000	0.001	0.000	0.000	0.000	—	−0.066	0.154
19	0.003	0.003	0.003	0.003	0.003	0.003	0.007	0.003	0.004	0.000	0.000	0.000	0.001	0.000	0.000	0.000	0.000	—	0.233
20	0.003	0.003	0.003	0.003	0.003	0.003	0.007	0.003	0.004	0.000	0.000	0.000	0.001	0.000	0.000	0.000	0.000	0.000	—

Note: The lower left triangle is the genetic distance, and the upper right triangle is the genetic differentiation index.

### 3.2 Genetic structure and haplotype analysis

The haplotype diversity of the rbcl and trnL-trnF sequences was calculated by DNAsp and showed that the rbcl sequence had a high haplotype diversity with 19 haplotypes detected individually with a haplotype polymorphism of 0.561. 9 haplotypes were detected in the trnL-trnF sequence with a haplotype polymorphism of 0.514. The haplotype analysis showed that 15 haplotypes were detected in the combined sequences, with a haplotype polymorphism of 0.544, a haplotype diversity variance of 0.00035, and a standard deviation of 0.019 (Table 3).

An analysis of the haplotypes of all populations in the combined sequence shows that populations 7 and 18 have the highest number of haplotypes. In terms of haplotype diversity, population 10 is the highest at 0.6. Populations 2, 3, 4, 5, 6, 8, 11, 12, 13, 14, and 20 have a single haplotype, and therefore all have zero haplotype polymorphism (Table 4).

**FIGURE 3:**
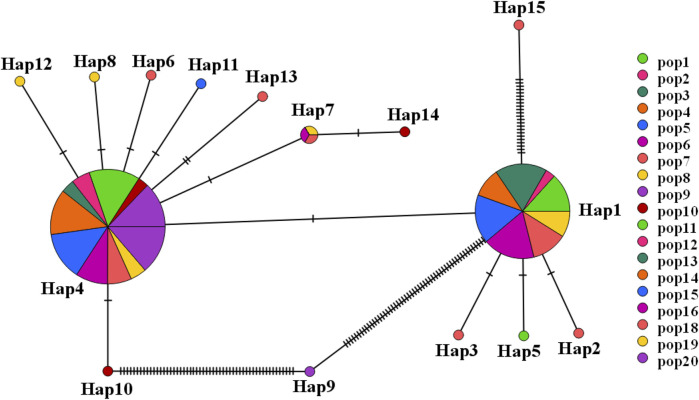
The network for haplotype based on combined sequence. (Different colors represent different haplotypes).

A haplotype network structure map of merged sequences between different geographical locations of C. squarrosa was constructed by the minimum-spanning method (Figure 3). Haplotypes are denoted by Hap. The merged sequences were divided into 15 haplotypes. Hap11, Hap6, Hap13, Hap12, Hap8, Hap10, Hap7 are directly linked to Hap4, while Hap14 is linked to Hap4 via Hap7. Hap2, Hap3, Hap6, and Hap15 are directly linked to Hap1, while Hap9 is special and may be linked to Hap4 by one or several mutations. And the NJ tree, the MP tree and the ML tree show similar results.

**FIGURE 4:**
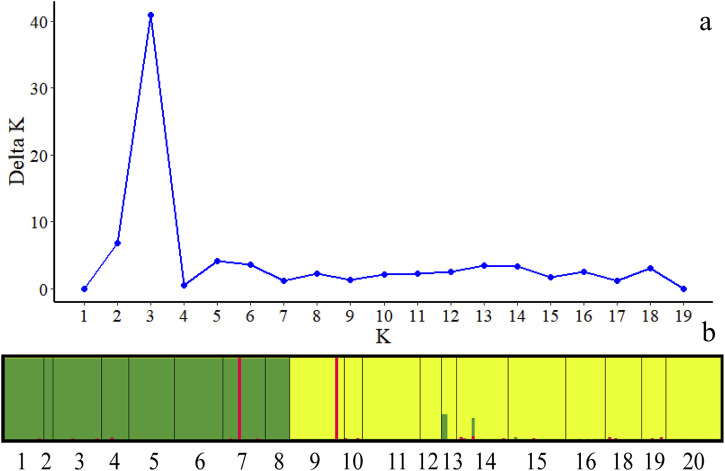
Bayesian model-based clustering STRUCTURE analysis of 234 individuals of *C. squarrosa*. **(A)** K values for different numbers of clusters (K); **(B)** Estimated population structure of 234 *C. squarrosa* individuals on K = 3. (In picture b, populations of the same color are clustered into one class).

The structure analysis suggested K = 3 as the optimal number of clusters based on the calculation of ΔK, which indicated that the 236 individuals from the 19 populations most likely belonged to three main genetic clusters (Figure 4A). Cluster I mainly included individuals from the western group, containing 1, 2, 3, 4, 5, 6, 7 and 8 populations, whereas Cluster II consisted of most individuals from the eastern group, including 9, 10, 11, 12, 13, 14, 15, 16, 18, 19 and 20 populations. However, some individuals with admixed genotypes in both groups indicated ongoing gene flow or weak genetic differentiation. There are also a very small number of individuals clustered together in a separate category (Figure 4B).

**FIGURE 5:**
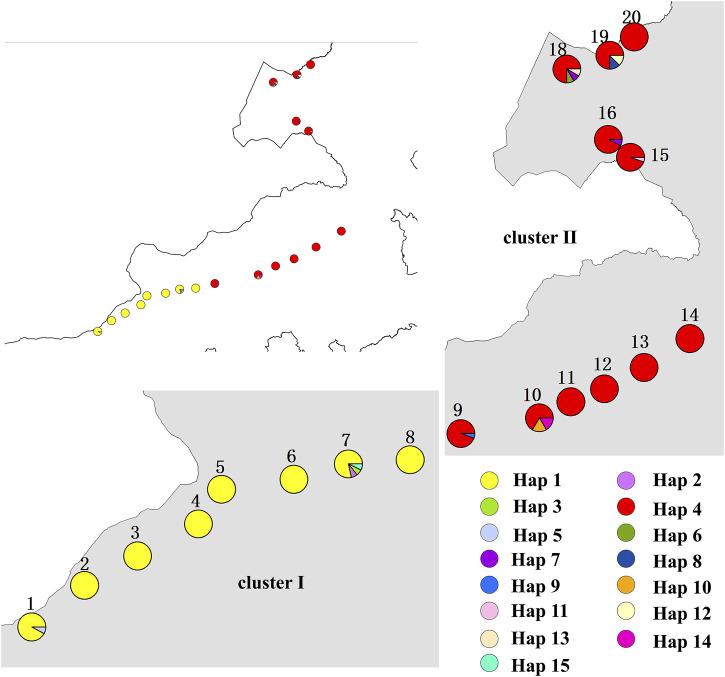
A map showing the sampled populations of *C. squarrosa* and the distribution of haplotypes. Pie charts show the proportions of the haplotypes within each sample. Haplotypes are indicated by different colors. (The location maps for clusterⅠ and clusterⅡ are enlarged versions of the top left image).

**FIGURE 6:**
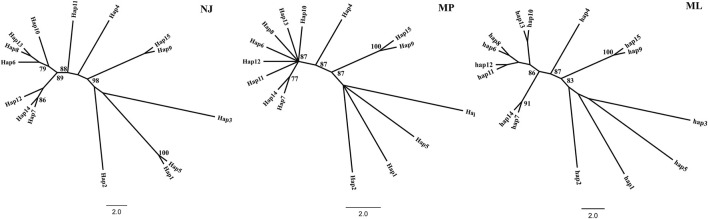
Phylogenetic relationship of the haplotype. Note: Different lowercase letters showed different haplotypes. The numbers at the nodes in the figure are confidence intervals.

To further demonstrate gene flow among different populations, we estimated frequency changes of dominant haplotypes at the combined sequence in other locations (Figure 5). The total number of haplotypes for *C. squarrosa* was 5 and 10 in the cluster I and cluster II groups, respectively. Among these haplotypes, Hap1 was widely distributed in cluster I, whereas Hap4 was widely distributed in cluster II, which indicates that these two haplotypes were favored in two groups. In addition, we found some distinct differences in haplotype frequency among C. squarrosa from different locations. Some environment-specific haplotypes could be found only in one location, such as Hap5, Hap2, and Hap3 in cluster I, and Hap4, Hap9, and Hap10 in cluster II. The NJ tree showed that the *C. squarrosa* accessions could be differentiated according to their geographic locations, including a cluster from Hap1、Hap2、Hap3、Hap5、Hap 9、Hap15, and another cluster from Hap4、Hap6、Hap7、Hap8、Hap10、Hap11、Hap12、Hap13、Hap14 (Figure 6). This clustering result is consistent with the clustering result in Figure 3.

To further ensure the accuracy of the clustering diagram STRUCUTRE 2.3 was used for Bayesian clustering analysis and based on the calculation of ΔK, the STRUCTURE analysis showed that K = 2 was the optimal number of clusters (Supplementary Figure S1). This suggests that the 15 haplotypes may belong to two major clusters and among them haplotype 9 and haplotype 15 clustered together which is consistent with the trend of the clustering results described above and justifies the reasonableness of our clustering results.

### 3.3 Correlation between genetic distance, geographic distance, and climate

**FIGURE 7:**
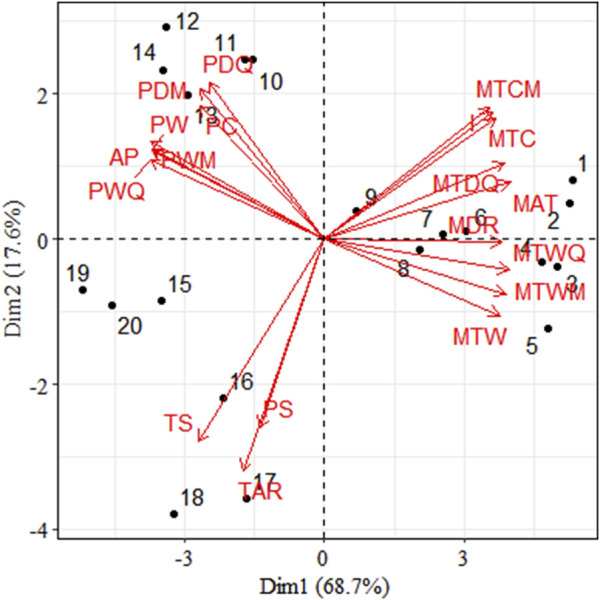
Principal component analysis (PCA) of environmental variables of 19 populations. (Note: MAT, Mean Annual Temperature; MDR, Mean Diurnal Range; I, Isothermality; TS, Temperature Seasonality; MTWM, Max Temperature of Warmest Month; MTCM, Min Temperature of Coldest Month; TAR, Temperature Annual Range; MTWQ, Mean Temperature of Wettest Quarter; MTDQ, Mean Temperature Driest Quarter; MTW, Mean Temperature of Warmest Quarter; MTC, Mean Temperature of Coldest Quarter; AP, Annual Precipitation; PWM, Precipitation of Wettest Month; PDM, Precipitation of Driest Month; PS, Precipitation seasonality; PWQ, Precipitation of Wettest Quarter; PDQ, Precipitation of Driest Quarter; PW, Precipitation of Warmest Quarter; PC, Precipitation of Coldest Quarter).

The results of the principal component analysis showed that PC1 (68.7%) not only divided the 11 temperature-related variables and 8 precipitation-related climatic variables into 3 clusters, except for seasonal temperature (TS), annual temperature range (TAR) and seasonal precipitation (PS), where precipitation and temperature were more clearly differentiated. Population 1 to population 9 is mainly influenced by temperature and shows positive correlation, population 10 to population 14 is mainly influenced by precipitation and shows positive correlation, population 15 to population 19 is mainly influenced by TST, AR and PS and shows positive correlation (Figure 7).

**FIGURE 8:**
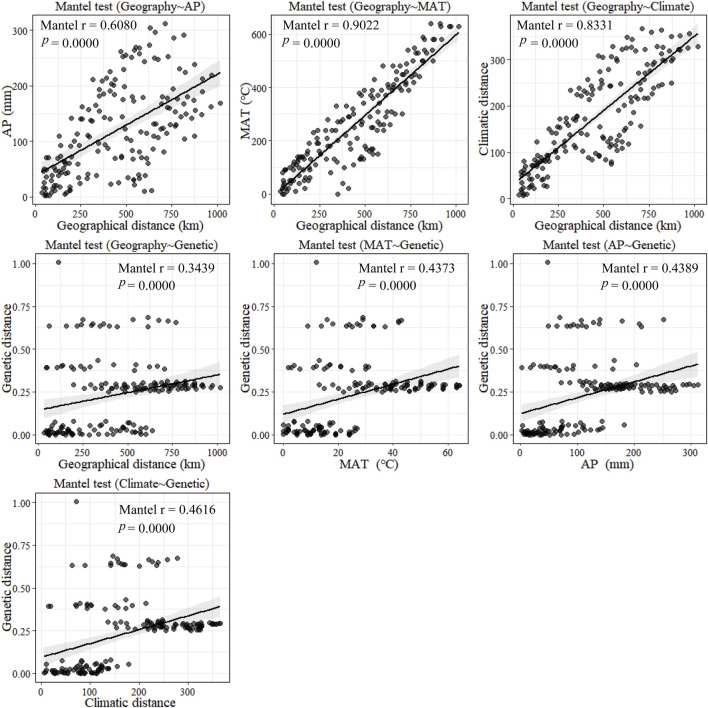
Plot of mantel tests for the correlation between genetic diversity parameter (Bray–Curtis distance) and explanatory distances (geographic and environmental distance) using Spearman’s coefficient.

**FIGURE 9:**
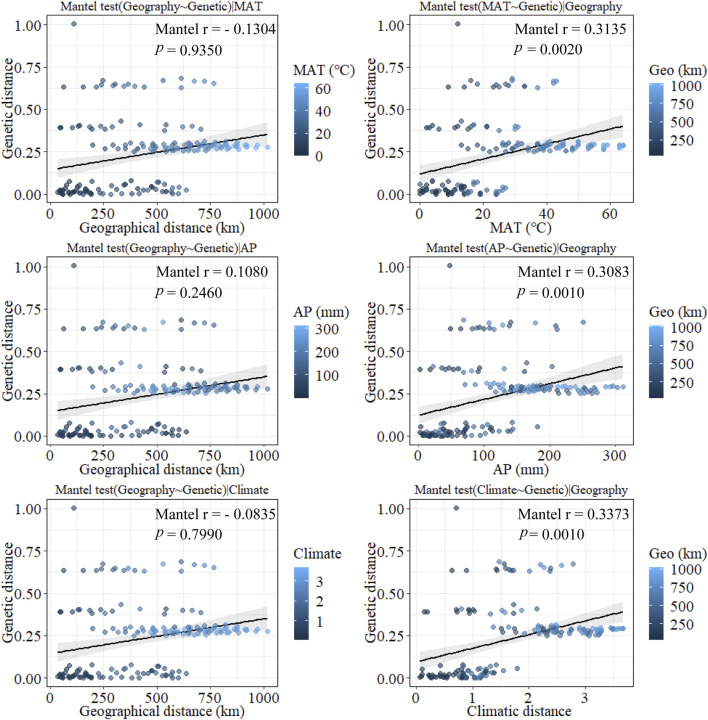
Plot of partial mantel tests for the correlation between genetic diversity parameter (Bray–Curtis distance) and explanatory distances (geographic and climatic distance) using Spearman’s coefficient. (This part of the test is a correlation test after excluding the effects of some of the certain factors such as MAT, AP, Climatic factors, and Geo factors separately).

According to the mantel tests, both climatic and geographic distance significantly affected genetic distance. Climatic distance had a more substantial effect on genetic distance than geographic distance. In addition, there was a significant correlation between geographic distance and ecological distance (Figure 8). The pure impact of climate on genetic distance was highly substantial according to the partial mantel test. While excluding climatic factors, the pure effect of geographical distance on genetic distance was insignificant (Figure 9).

Furthermore, the mantel tests of annual precipitation and annual mean temperature showed that annual precipitation had a more significant impact on genetic distance parameters than annual mean temperature. According to the partial mantel test, the pure effect of annual precipitation and mean annual temperature on genetic distance were all significant when geography was excluded. In addition, the geographical distance was not correlated with climatic distance when the effect of mean annual temperature was not considered. In contrast, geographical and climatic distance were significantly correlated when the effect of annual precipitation was excluded. These results suggest that climate has a more significant impact on genetic distance than geographical distance does on genetic distance and that among climatic factors, annual precipitation has a more substantial effect on genetic distance than mean annual temperature.

## 4 Discussion

### 4.1 Genetic diversity

We investigated genetic diversity using sequencing data for the rbcl and trnL-trnF chloroplast genes of *C. squarrosa*. Tajima’s D values and mismatch distributions suggest that *C. squarrosa* has undergone population expansion, meaning that populations adapt well to the environment in their area. AMOVA analysis revealed some genetic variation within C. squarrosa populations, but genetic variation was mainly between populations (FST = 0.5917). But the genetic variation was mainly between populations (FST = 0.5917). Genetic differences between the populations may have been caused by the limited gene flow due to the species’ limited seed dispersal (Liu et al., 2016).

Haplotype diversity of 0.544 and nucleotide diversity of 0.00096 in C. squarrosa indicated low genetic diversity. Specific characteristics of the species influence the genetic diversity of plants. The low level of genetic diversity may be due to the reproductive behavior of *C. squarrosa* is a parthenogenic pollinator, with both self-pollination and open pollination present in the same single plant, which allows *C. squarrosa* to exhibit high levels of inbreeding and, over time, produce too many pure congeners, leading to a loss of its genetic diversity, which may have led to low levels of genetic variation in some populations (de Groot et al., 2011).

Genetic diversity varies between populations of C. squarrosa. Populations 7 and 18, which have the highest genetic diversity, contain four haplotypes, while populations 2, 3, 4, 5, 6, 8, 11, 12, 13, 14, and 20 contain only one haplotype. In some populations, low genetic diversity at the cpDNA level is always related to demographic events. The haplotype network suggests that the frequency of haplotype 9 may indicate an expansion event. Therefore, demographic events have played an essential role in shaping the genetic diversity of *C. squarrosa*. It has been suggested that potential refugia or centers of diversification should have high genetic diversity, while recolonized sites exhibit lower genetic diversity. Our study found the highest genetic diversity in population 7, suggesting that this population may be a refuge or center of diversification for C. squarrosa (Liu et al., 2016).

### 4.2 Genetic structure

STRUCTURE analysis confirmed that the populations of *C. squarrosa* species clustered ecogeographical, generally in line with their bioclimatic regions, because those sharing the same climate environment grouped in the same cluster, irrespective of their geographic distances (Hamasha et al., 2013). Further discussion in conjunction with the main influences derived in PCA based on precipitation and temperature changes. Typical grasslands have an annual precipitation of 200–400 mm and an average annual temperature of −1.0°C–6.1°C, while desert steppes are the driest type of steppes in the steppe to desert steppes, with an annual precipitation of 150–280 mm and an average annual temperature of 2.6°C–7.8°C (Hamasha et al., 2013). Populations living in the same climatic environment can be divided into the same clusters. The two clusters belong to different climatic zones (Wu et al., 2010). Consequently, variability in precipitation and temperature impose fluctuating selection pressures between other bioclimatic regions, influencing phenological patterns such as flowering time and timing of seed germination. This may also increase the genetic differentiation of populations in the two climatic zones (Xie et al., 1992). Such differences in reproductive phenology may lead to partial reproductive isolation, favoring population adaptation to different habitats and neutral genetic differentiation (Ping et al., 2013). Differences in such demographic processes may produce patterns of genetic variation that resemble patterns due to natural selection (Rouger and Jump, 2015). Population genetic structure developed under restricted gene flow cannot be distinguished from population structures built by spatially heterogeneous samples (Cox et al., 2016). Thus, genetic differentiation between bioclimatic regions may have been caused by local adaptation rather than solely by demographic processes (Borokini et al., 2021). Hence, the genetic structure of plant populations is not randomly distributed but is associated with ecogeographic factors (Hamasha et al., 2013).

Variations in humidity and temperature among habitats can cause deviation in flowering phenology and consequently limit gene flow, which leads to a high probability of reproductive isolation. Temperature is an essential factor influencing population differentiation and potential adaptation, and temperature can affect populations through life history traits, seedling tolerance, and temperature requirements for initial growth (Al-Gharaibeh et al., 2017). *C. squarrosa* is more sensitive to environmental changes, and temperature and precipitation may affect the phenology of *C. squarrosa*, particularly the timing of flowering, thus affecting the exchange of genes between populations. Therefore, we assume that the differences in drought and temperature between the phytogeographic regions isolated the *C. squarrosa* populations’ climatic and shaped their genetic structure (Zhang et al., 2014). Low rainfall resulted in limited seed dispersal around the mother plant, creating an adaptation of the genetic system to minor population conditions and a restricted gene flow rate which increases genetic differentiation among populations (Cox et al., 2016). High levels of genetic differentiation between plant populations have shaped the genetic structure of *C. squarrosa* (Kabiel et al., 2013).

### 4.3 Climatic effects

A significant positive correlation between genetic distance and geographical distance was found in our study, with the genetic distance increasing with the geographical distance from west to east (Garot et al., 2019). Wright (Wright: Genetics 28:114–138, 1943) proposed that given limits on dispersal and in the absence of selection, drift would cause populations to become more differentiated at greater distances (Sexton et al., 2014). In the study of Volis, there was no significant correlation between the genetic distance and the geographical distance if the environmental choice caused genetic differentiation (Wu et al., 2010; Al-Gharaibeh et al., 2017). In addition, *C. squarrosa* is a parthenogenetic pollinator and our results suggest that selfing may predominate in the mating system of *C. squarrosa*. In contrast to other long-lived, wind-pollinated, predominantly heterozygous gymnosperms, *C. squarrosa* is more limited in its ability to spread seeds and therefore has difficulty spreading to other regions and restricted gene exchange leads to increased genetic distances with increasing geographical distance (Buer et al., 2022).

Genetic distance and precipitation were significantly and positively correlated with temperature in this study (Zhao et al., 2022). They remained significantly correlated after excluding the effect of geographical distance, and genetic distance increased with increasing temperature and precipitation (Cui D. et al., 2021). Different precipitation and temperature gradients had different effects on *C. squarrosa*, and this effect led to a mismatch in flowering time between individual *C. squarrosa* populations, i.e., non-random mating occurred, which led to an increase in the genetic distance between populations (Sexton et al., 2014). In addition, different precipitation and temperature gradients exert strong differential selection pressures on *C. squarrosa* (Wu et al., 2010). With limited gene flow, *C. squarrosa* populations can develop various adaptations, leading to differences between genetic distances (Lin et al., 2006).

## 5 Conclusion

The degree of genetic diversity in *C. squarrosa* is low and structure analysis reveals the presence of two genetic clusters. We suggest that geographical distance and environmental factors may explain the genetic differentiation between these populations, that the main factors shaping the genetic structure of the populations are annual precipitation and mean annual temperature, and that differences in precipitation and temperature may affect the adaptability of *C. squarrosa* and the phenological period. Exploring the effects of climatic factors on the genetic variation of *C. squarrosa* under a large scale gradient, exploring the effects of climatic factors on the genetic variation of *C. squarrosa* can not only provide information on the genetic variation of C. squarrosa, but also provide a theoretical basis for the genetic adaptation mechanism of grass plants. The gene sequences measured by our experiments can be applied to the training phase of machine learning models such as multi-information fusion techniques, and effective learning models will be identified for application in drug targeting studies. (Zhao et al., 2023).

## Data Availability

The datasets presented in this study can be found in online repositories. The names of the repository/repositories and accession number(s) can be found in the article/Supplementary Material.

## Supplementary material

SUPPLEMENTARY FIGURE S1
Bayesian model-based clustering STRUCTURE analysis of 15 haplotype of the merged sequence. **(A)** K values for different numbers of clusters (K); **(B)** Estimated clustered structure of 15 haplotype on K = 2. (In picture b, haplotype of the same color are clustered into one class.
