# Supplementary figures and images for: Climatic factors, but not geographic distance, promote genetic structure and differentiation of Cleistogenes squarrosa (Trin.) Keng populations
Source: Front Bioinform. 2024 Nov 13;4:1454689. doi: 10.3389/fbinf.2024.1454689 (PMC11599168; doi:10.3389/fbinf.2024.1454689)

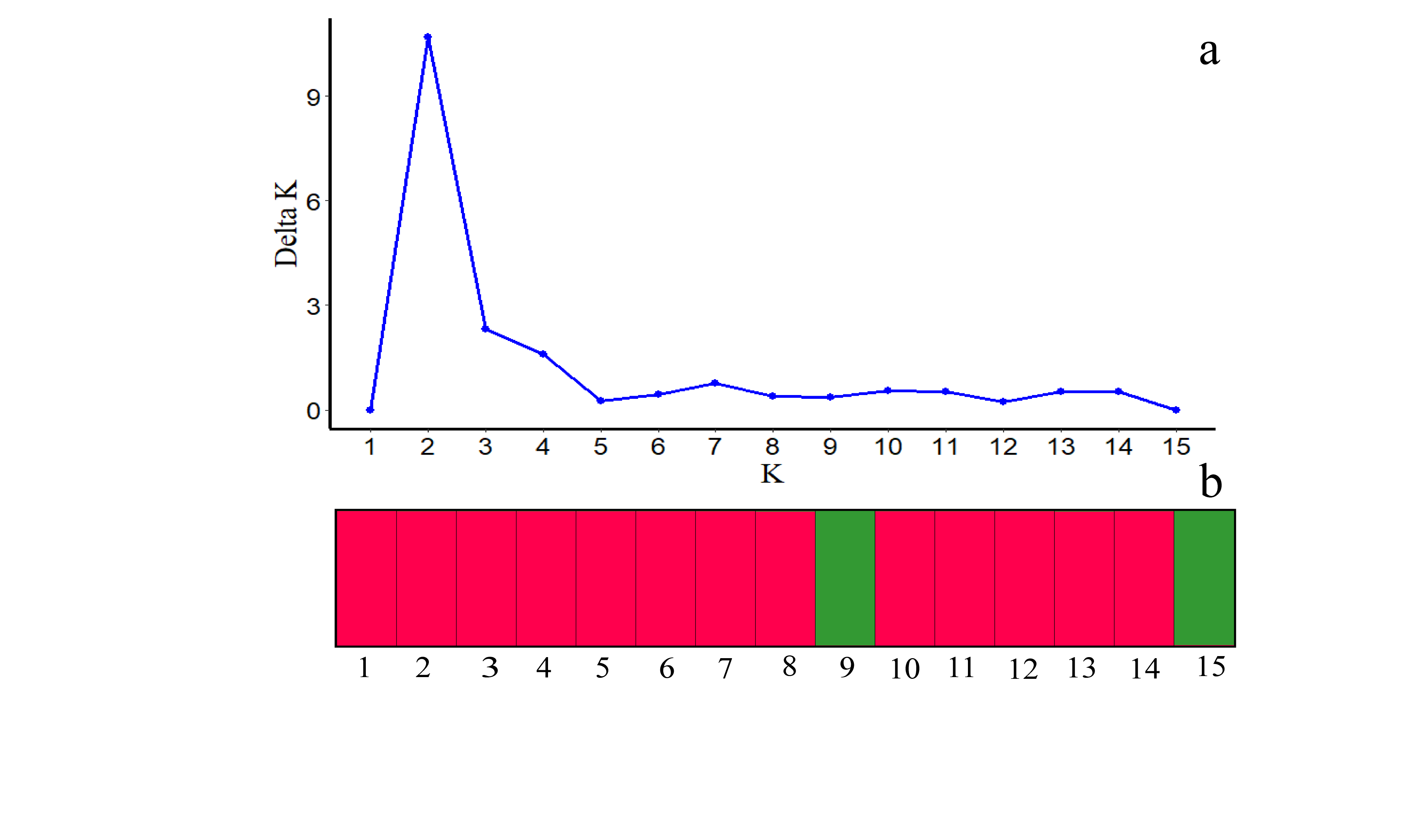

Supplement: Supplementary file 1 [file Image1.tif]
